# Direct evidence of increased natural mortality of a wild fish caused by parasite spillback from domestic conspecifics

**DOI:** 10.1098/rspb.2022.1752

**Published:** 2023-01-25

**Authors:** Knut Wiik Vollset, Robert J. Lennox, Helge Skoglund, Ørjan Karlsen, Eirik Straume Normann, Tore Wiers, Elisabeth Stöger, Bjørn T. Barlaup

**Affiliations:** ^1^ Climate and Environment, NORCE Norwegian Research Centre, Laboratory for Freshwater Ecology and Inland Fisheries, Nygårdsgaten 112, 5008 Bergen, Norway; ^2^ Norwegian Institute for Nature Research, Høgskoleringen 9, 7034 Trondheim, Norway; ^3^ Institute of Marine Research, Nordnesgaten 50, 5005 Bergen, Norway

**Keywords:** sea lice, *Lepeophtheirus salmonis*, salmonid, marine survival, conservation

## Abstract

Parasite spillback from domestic animals can distort the balance between host and parasites in surrounding wildlife, with potential detrimental effects on wild populations. In aquatic environments, parasite spillback from aquaculture to wild salmon is one of the most contentious sustainability debates. In a 19 year time series of release group studies of Atlantic salmon, we demonstrated that (i) the effect of subjecting out-migrating salmon smolts to parasite treatment on marine survival has been reduced over a time, (ii) the relation between salmon lice levels in the out-migration route of the salmon and effect of treatment against the parasite is weak, but also (iii) the return rates in both treated and untreated groups of salmon are negatively correlated with salmon lice levels, and (iv) returns of wild salmon to the region are similarly negatively correlated with salmon lice levels during the out-migration year. Our study suggests that salmon lice can have a large effect on wild salmon populations that is not revealed with randomized control trials using antiparasitic drugs. This should be better accounted for when considering the impacts of farms on wild salmon populations.

## 1. Introduction

The collateral effect of disease spread from farmed to wild animals is one of several challenging environmental impacts of intensive farming on wildlife [1]. Such impacts occur when native farmed animals or introduced farmed species serve as hosts for parasites that also infest sympatric wildlife that is vulnerable to the pathogen. Documenting the population effect of this mechanism on wildlife is challenging because it requires disentangling the natural dynamics of the host–parasite system and the role of the added hosts to the ecosystem [2].

**Figure 1.**
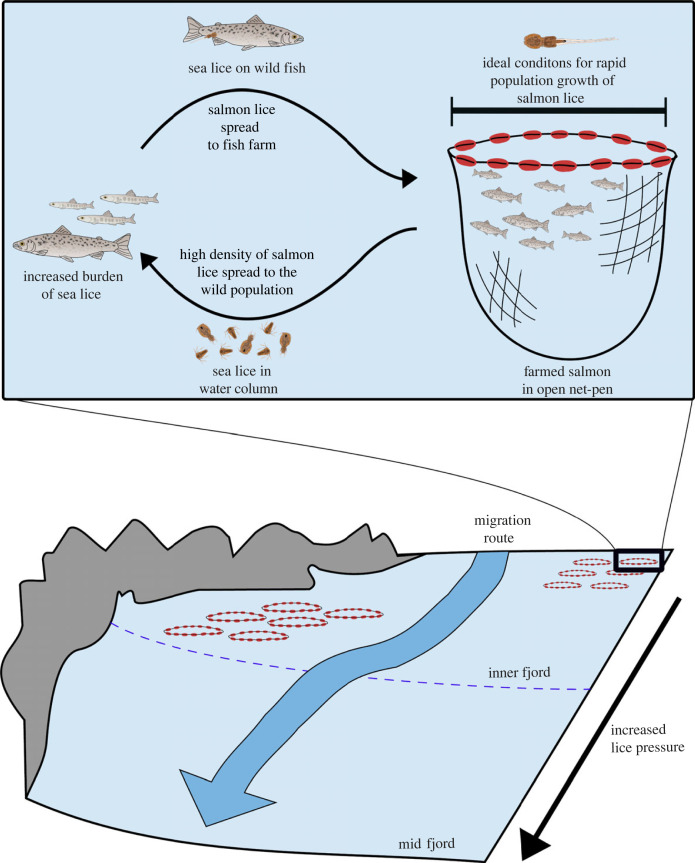
Illustration of parasite spillover–spill back dynamic between fish farms and wild salmonids. Credit: Cecilie Iden Nilsen.

The proliferation of the endemic parasitic crustacean *Lepeophtheirus salmonis*, or salmon louse, in fish farms is perhaps the foremost example of intraspecific parasite spillback in aquatic environments, i.e. where a local species is farmed, increasing the total host abundance for an endemic parasite in the environment [1] (figure 1). Atlantic salmon (*Salmo salar*) infested with lice incur damage to the skin, which may lead to pathological responses in the host when numbers are unnaturally high, ultimately resulting in death if untreated [3–5]. The presence of fish farming has repeatedly been linked to epidemic outbreaks of salmon lice on wild salmonids [6,7]. Consequently, salmon lice parasitism of wild Atlantic salmon, sea trout (*Salmo trutta*), and sea-run Arctic char (*Salvelinus alpinus*) post-smolts is one of the major environmental concerns for wild salmonids in countries with intensive salmon farming [8–10]. Notwithstanding, quantifying the exact population effect has remained a topic of controversy [11,12], with reports of large to minor effects on wild salmon [13–15].

The main source of data about the effects of sea lice *in situ* on hosts is randomized control trials (RCTs; [16,17]). These trials involve releasing groups of tagged hatchery-reared salmon smolts where half of the salmon are treated with an antiparasitic drug while the other half is left untreated. Assuming that the treatment protects the salmon smolt from lice infection and does not affect the physiology or survival, such trials should provide the data to estimate the impacts of salmon lice on the marine survival of smolts. Effect sizes have been reported to vary from on average 11 to 39% reduction in return rates of adult salmon [12,14,16–18], but also as only approximately one percentage point of the variation of the total marine survival [11].

Some have raised concerns about how efficient these antiparasitics are at shielding out-migrating salmon smolts [14,19]. For example, studies have shown that there is variation in the amount of drug taken up through oral administration within groups of treated fish [20], and that the treatment itself has been shown to reduce the survival of salmon smolts as they migrate to sea [21]. But perhaps of most concern is that the treatment (e.g. emamectin benzoate) has been shown to be decreasingly effective owing to increasing levels of resistance among salmon lice adopted via evolution of the physiological system [22,23]. In several studies, the effect size of treatment has been decreasing over time [16,17], which has been used as an argument that the effect of parasite spillback has been curtailed by management actions, with some voicing opinions that management is too strict. However, in the same areas this has counterintuitively been paralleled with an increasing parasite load on wild fish during surveillance [6], and also an increased modelled infestation pressure on wild fish [24]. There are, therefore, lingering questions about whether these contradictory patterns suggest that salmon lice are not as detrimental as earlier studies have suggested, or that RCTs are underestimating the effect of salmon lice on wild salmon survival.

One of the longest time series RCTs has been conducted using fish from the River Vosso, a major catchment in western Norway where trials were started in 2000. Simultaneously, salmon lice surveillance has been conducted on sea trout along the out-migration route of the salmon smolts since 2009, providing a unique proxy for estimating temporal variation in lice infestation pressure. These unique time series yield the opportunity to model the relationship between salmon lice treatment effects and lice infestation pressure, and test whether the antiparasitic treatment is reducing the effect of salmon lice on the return rate. In this study, we explored (i) the temporal trend in effect of salmon lice treatment, (ii) whether the effect of treatment correlates with infestation pressure, (iii) whether interannual return rates of treated and untreated hatchery fish correlate with infestation pressure, and finally, (iv) whether return rates of wild salmon in the region correlate with infestation pressure.

## 2. Material and methods

### (a) Study site

The Vosso River is situated in the inner part of the Osterfjord system in Nordhordland on the west coast of Norway. The Vosso is one of Norway's most renowned salmon rivers, famous for its particularly large salmon [25]. The salmon population in Vosso collapsed around 1990, and since 2000 hatchery-produced salmon smolts have been released in the river or towed in transport tanks to increase spawning abundance, and to provide an experimental basis to study potential survival bottlenecks on out-migrating salmon smolts. Survival from releases of hatchery fish in freshwater has been very low (approx. 0–0.5%), while fish towed 15–105 km from the river mouth have had higher, albeit relatively low, survival (approx. 0.5–4%).

### (b) Release groups of salmon

**Figure 2.**
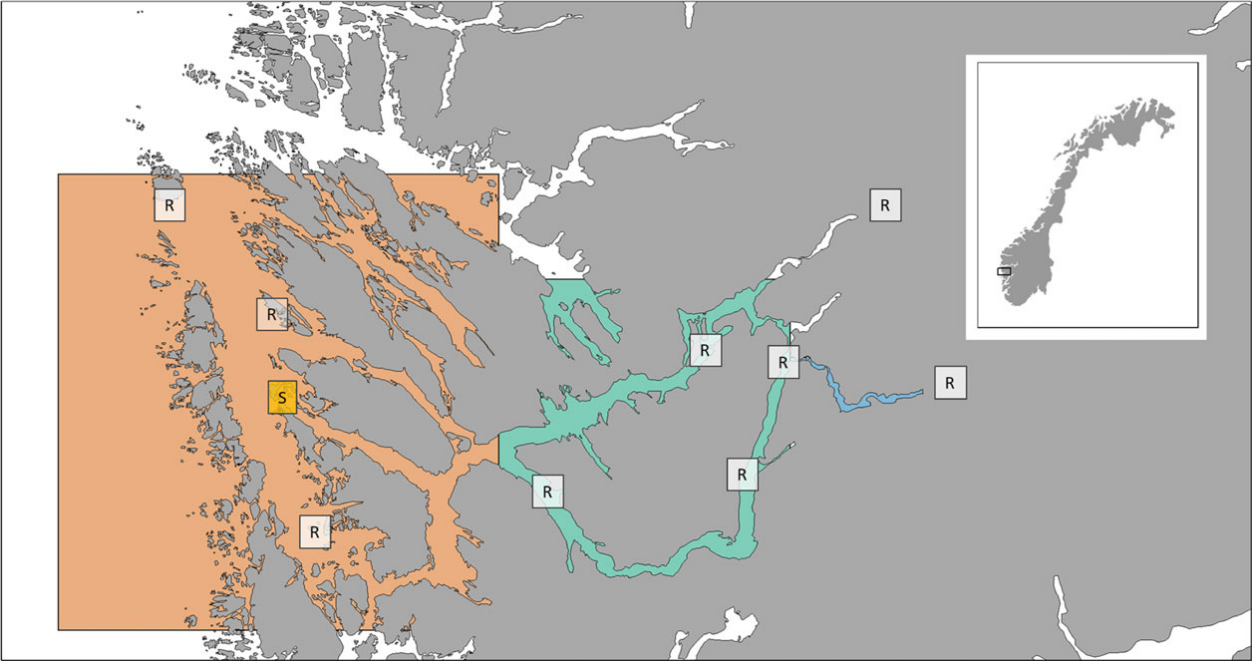
Map showing the different release areas, with release category coloured as blue = river, green = inner fjord, red = outer fjord. R marks various release locations, while S indicates (in yellow) location of salmon lice surveillance.

**Figure 3.**
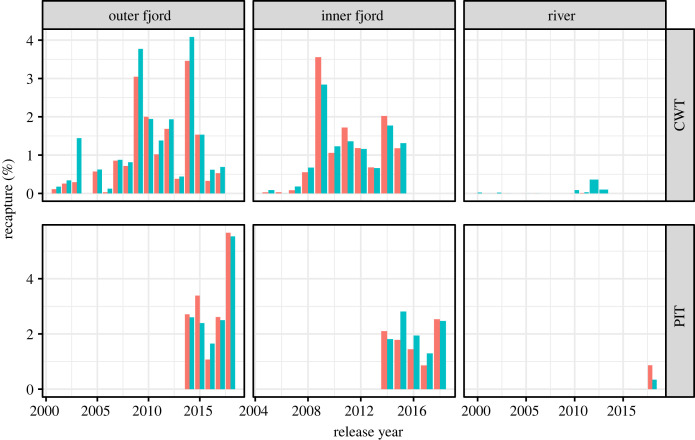
Overview of return rates from release group studies, where red bars show recapture rates of control group and blue show recapture rates of fish treated with antiparasitic treatment. The groups have been summed within years and divided into release site and tag type.

Each release group has been used as an RCT of hatchery-reared salmon smolts, where half of the fish have been treated with an antiparasitic drug. This method has been described in various other publications [13,16,17,26]. The method involves rearing salmon eggs originating from the national Gene Bank to smolt size in hatchery facilities for 1 year, and then treating the salmon smolts with fish feed pellets coated with emamectin benzoate (SLICE^®^). These fish are then released into the river or transported in tanks or mobile net-pens further out into the fjord before release. The fish are tagged with either coded wire tags (CWTs; years 2000–2017) or passive integrated transponders (PITs; 2015–2019) so that it is possible to identify them as they are recaptured or registered on an antenna upon their return as adults. In a few trials, another antiparasitic treatment (Substance EX) has been used, but in most cases the emamectin benzoate has been the only available treatment. Releases of hatchery-reared salmon in freshwater have not been successful in this system, i.e. very few fish have returned from any group released in the river, lakes or estuary of the Vosso. Since the release groups are also a part of a restoration effort of the Vosso salmon, some years fish have only been released in the fjord. There has been some variation in the release sites in the fjords, but for the purpose of this study we group the release groups into those that have been released in the outer fjord (70–105 km from the river mouth), in the inner fjord (15–70 km from the river mouth), and in freshwater (approx. 10 km upriver to 15 km from the river mouth). The two most prevalent locations are at Manger (WGS84; 60.63918° N, 4.92149° E) and Arna (WGS84; 60.50812° N, 5.37777° E) (figure 2). Recaptures (including antenna registrations) in these groups have varied from 0% to more than 4% throughout the time series (figure 3).

### (c) Sea lice surveillance

Sea lice surveillance on sea trout has been conducted at Herdla, the northern peninsula of the island of Askøy (WGS84; 60.568972° N, 4.963010° E) since 2009. Here, trout have been caught using a trap-net that has been developed specifically to capture and treat trout while minimizing sea lice loss during handling [19]. From an earlier study by Vollset *et al*. [6], it has been shown that the lice numbers on sea trout at this site correlate with the infestation pressure of fish farms in the outer region of the fjord. This area is also one of the largest fish farm zones with coordinated production and fallowing in the outer fjord system where all the released salmon smolts must migrate (see [6]). This is also the area where surface salinity layers permit salmon lice to overlap with out-migrating salmon smolts [16,17].

The number of trout caught during the monitoring season has varied with weather conditions, sampling intensity and number of traps operated. The way that trout are handled is described in more detail in Vollset *et al*. [6], but in brief, the trap chambers are checked daily, and individual trout are transferred from the trap using a hand-held dip net and are either euthanized and placed in a zip-lock bag or transported in a large bucket with aerated water to land. Euthanized samples are kept cold, frozen when they reach land, and later thawed and counted in the laboratory, while live samples are counted after being sedated with a half dose (0.05 g l^−1^) of MS222 and then assessed for salmon lice in a high-contrast bucket using a headlamp by trained personnel. Since 2015 the sea lice surveillance at Herdla is also operated as a part of the Norwegian national sea lice monitoring programme.

We aimed to use a standardized time period from which to assess sea lice numbers on sea trout that can be representative of the lice infestation pressure from when the tagged hatchery salmon smolts are released. When counting sea lice on sea trout, the most observable lice are large chalimus and mobile stages, while recently attached copepods are more likely to be missed. Therefore, we use total lice counts on sea trout from day of year 135 to 165 as an assessment of the infestation pressure the salmon smolts must experience. This corresponds to approximately 15 May to 15 June, and is based on a study on progression rate of salmon smolts from hatchery smolt in this area [16,17]. To account for the fact that larger fish will attract more parasites, we use number of parasites per gram of fish per individual and average data to get one index per year. This method is expected to provide a fair index of interannual variation of the infestation pressure.

### (d) Statistical analysis

#### (i) Risk ratio analysis of whole time series

First, we analysed the effect size of treatment as a risk ratio (RR). This is similar to the standard metaregression used in Borenstein *et al*. [27], where the response variable is the natural logarithm (ln) of RR, where RR is calculated as

$$\mathrm{RR}=\frac{\left(\mathrm{NRt}/\mathrm{NTt}\;\right)}{\left(\mathrm{NRc}/\mathrm{NTc}\right)},$$

where NRt is number of adult recaptures from treated group, NTt is total number of treated released hatchery smolt, NRc is number of adult recaptures from untreated group and NTc is total number of untreated released hatchery smolt. In the regression each ln RR value is weighted based on:

pwr=1/(1/(NRt)−1/(NTt)+1/(NRc)−1/(NTc)).

With these values, a linear model can be built,

ln⁡RR∼1+(1|release_year), weights=pwr,

where ln RR is the log-transformed risk ratio and release_year is defined as a random effect. For the sake of comparison, we compared the estimate of this model with a meta-analysis as explained in Borenstein *et al*. [27] using the ‘meta’ package in R [28]. This linear model can be extended to a metaregression where various explanatory variables can be included instead of just the intercept as in the above model. For this analysis, we built one model for the entire time series with release site (river, inner fjord, outer, fjord) as an explanatory variable, and a second for the time period from 2009 to 2018, when estimates of infestation pressures were available (as explained above), where release site and infestation pressure and their interaction were included. The full model was

ln⁡RR∼release_place ∗ lice_trout+(1|release_year), weights=pwr.

#### (ii) Return analysis of time series where sea lice infestation pressure has been conducted

Although RCTs will isolate the effect of treatment, they do not give insights into how survival varies among years or estimate how much of the variation in survival is described by the treatment effect. This is particularly poignant in a system where there is some debate as to whether the treatment intervention increases marine survival or not, and whether the treatment shields the salmon smolts from lice. The goal of this analysis was to evaluate whether return rates of hatchery-reared salmon were negatively correlated with infestation pressure, and whether this negative correlation was less prominent for groups of salmon that were treated with sea lice. A binomial model where the response variable was the number of recaptured as a proportion of the number of released hatchery salmon smolts was, therefore, built where the model was

$$\begin{array}{rl} & \mathrm{lmer}(\mathrm{NR}|\mathrm{NT})∼\mathrm{lice}\_\mathrm{trout}\;*\;\mathrm{treatment}\;+\;\mathrm{lice}\_\mathrm{trout}\;*\;\mathrm{release}\_\mathrm{place}\;\\ & +\mathrm{tag}\;(1|\mathrm{release}\_\mathrm{year}),\;\mathrm{family}=\mathrm{binomial}, \end{array}$$

where NR is the number of adults recaptured, NT is the number of hatchery smolts released, lice_trout is the average number of lice per gram of trout during day of year 135–165 of lice surveillance, 'treatment' a factor defining whether the fish were treated or not, release_place is either river, inner fjord or outer fjord, 'tag' is either PIT or CWT tags, and release_year is year of smolt release.

#### (iii) Correlation of infestation pressure and return rates assessed by spawning count

To assess whether infestation pressure correlated with the number of returning wild fish, data from spawning count from the nearby Dale and Ekso rivers were extracted. Spawning count data are not available for Vosso directly because water clarity is too low and two lakes in the catchment obstruct effective counts. However, the Dale and Esko rivers are annually assessed using spawning counts, as explained in Skoglund *et al*. [29]. Size categories are defined as 1–3, 3–7 and greater than 7 kg, which are the size categories that are likely to approximate to 1SW (salmon returning after one sea winter), 2SW (salmon returning after two sea winters) and MSW (salmon returning after more than 2 years at sea). These size categories are assessed visually and are likely to have some observation error [29]. In addition, hatchery fish are identified by their lack of adipose fin. By using 1SW and 2SW data it was possible to get an index of adult returns over the time series as follows:

$$\mathrm{RI}=1SW_{t-1}+2{\mathrm{SW}}_{t-2},$$

where RI is the return index, 1SW*_t_*_−1_ is the number of 1SW observed the year after and 2SW*_t_*_−2_ is the number of 2SW observed 2 years after release as smolts. Correlation between RI and infestation pressure was assessed with the Pearson's product–moment correlation using the cor.test function in R.

## 3. Results

### (a) Temporal pattern in salmon lice effects

**Figure 4.**
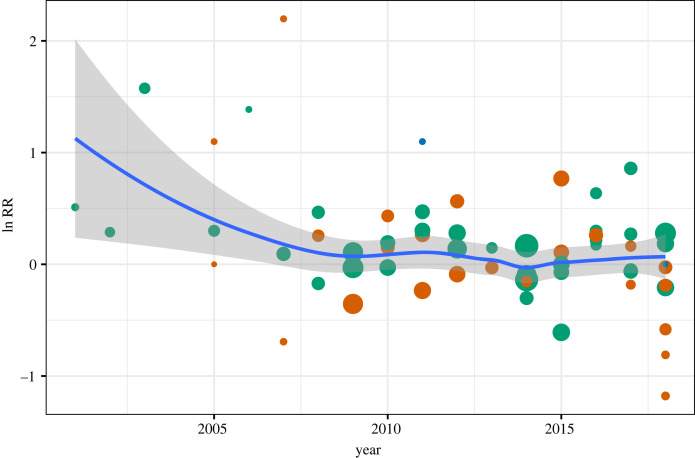
Temporal trend in effect size of treatment against salmon lice plotted as natural log of risk ratio (ln RR). Green is for the outer fjord, red for the inner fjord and blue for the river. The size of the dots corresponds to the weight of each treatment in the meta-analysis (pwr). The blue line with grey envelope is a loess smoother with 95% confidence interval.

In total, the dataset included 88 paired release groups over a period of 19 years (2000–2018), numbering 869 114 tagged and released smolts and 5839 adult recaptures. Among these, 78 groups were tagged with CWTs and 10 groups were tagged with PITs. Recapture rates in the groups varied from 0% in some release groups in the river at the beginning of the time series to 3.7% in the release group in the outer fjord in 2009. A random effect meta-analysis gave an overall estimate of 1.1006 (1.0194; 1.1883) RR and was significant (*Z* = 2.45; *p* < 0.05), meaning that treated fish were overall 1.1 times more likely to be recaptured as adults compared with control fish. The same estimate from the time period 2000 to 2008 was 1.4763 (1.0208; 2.1351) RR (*p* < 0.05), whereas from 2009 to 2019 gave an estimate of 1.0699 (0.9940; 1.1517) RR and was not significant (*p* = 0.072). This clear temporal pattern in effect size can also be seen in figure 4, where the effect declined and became indistinguishable from 0 on average. There was large and significant heterogeneity (*Q* = 120, *p* < 0.01) in the meta-analysis, with *τ*^2^ = 0.038 and *I*^2^ = 51.1%. This large heterogeneity can also be seen in figure 4, with a large variation in RR among groups.

### (b) Infestation pressure on sea trout

**Figure 5.**
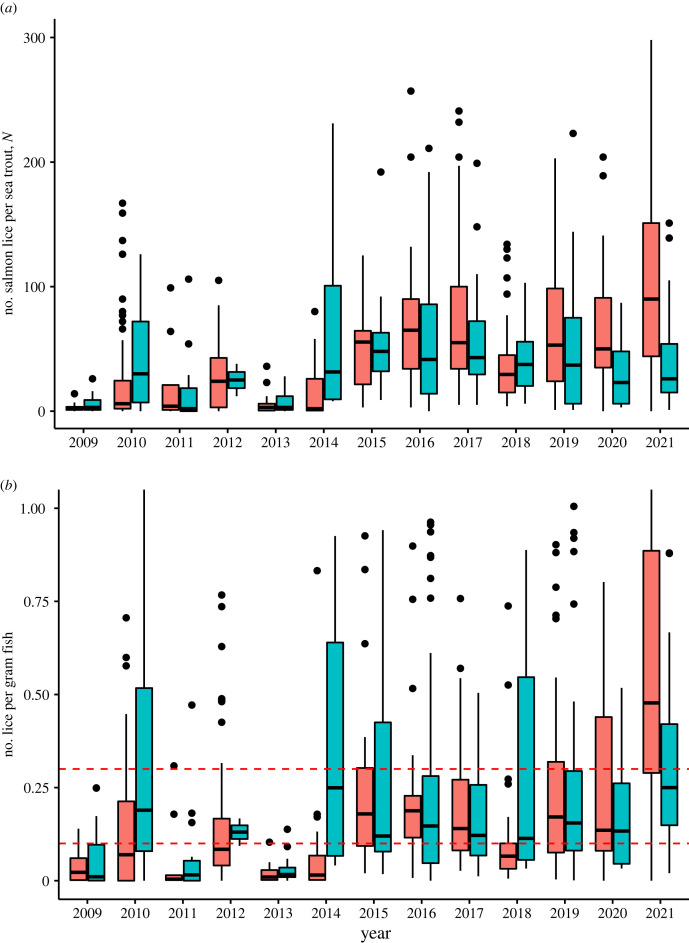
Boxplot of infestation levels on sea trout in the out-migration route of salmon in Nordhordland captured in a trap-net at Herdla, where blue indicates data from the last two weeks of May, while red indicates first two weeks of June. The box encloses the middle half of the sample, the midline indicates the median, while the lines extending from the box indicate the range of values excluding outliers (which are indicated by circles). (*a*) The total number of lice, (*b*) the number of salmon lice per gram fish weight. Red dashed lines indicate 0.1 and 0.3 lice per gram fish weight, which correspond to starting physiological effects and potential lethal levels of lice as described in Taranger *et al*. [30].

Infestation levels on sea trout varied throughout the time series, from low (e.g. 2011; avg. lice number = 2.5, median lice per gram = 0.005) to high (e.g. 2016; avg. lice number = 46; median lice per gram = 0.16). In figure 5, we have plotted the number of lice per gram of fish used in the analysis. For illustration purposes, we have divided the plot into May and June. Note that the time series continues to 2021 (the most recent datapoint in the time series) whereas the dataset on release groups is only updated to 2018. Since 2015, the lice levels have, with the exception of May 2018, had a median value above 0.1 lice per gram of trout.

### (c) Effect of infestation pressure on effect of treatment

**Figure 6.**
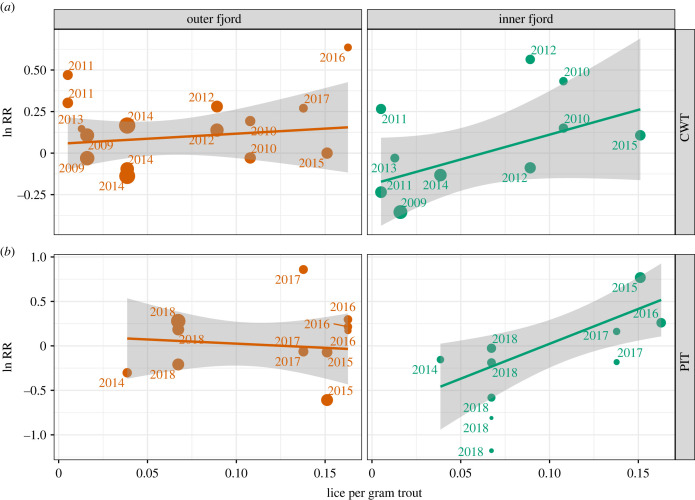
Risk ratio (RR) of treatment effect in released hatchery smolts plotted against the average number of salmon lice per gram of trout surveyed in the fjord. The size of the dots corresponds to the weight of each treatment in the meta-analysis (pwr). (*a*) Data from fish tagged with coded wired tags (CWTs); (*b*) data from fish tagged with passive integrated transponders (PITs). The lines and grey envelopes show linear fit to the data with 95% confidence interval.

There seemed to be no meaningful effect of the number of lice per gram of sea trout on the efficacy of prophylactic treatment in groups released in the outer fjord (table 1; figure 6). However, there was an increasing RR for release groups in the inner fjords, suggesting that release in the inner fjord amplified treatment effects. For river release groups there were so few fish that returned that it was not possible to calculate RR with a meaningful variance, and the data were, therefore, not included in the final model.

**Table 1.** Model coefficient of final model of risk ratio of treatment against salmon lice. lpg, number of lice per gram of trout.

predictor	model of risk ratio of treatment		
	estimate	CI	*p*
(intercept)	−0.26	−0.50 to −0.02	0.032
lpg	0.34	0.05–0.64	0.022
lpg: inner fjord	3.84	1.15–6.53	0.006
lpg: outer fjord	−4.09	−7.40 to −0.78	0.017
no. observations	47		
*R*^2^/*R*^2^_adjusted_	0.170/0.112

### (d) Effect of infestation pressure on return rate

**Figure 7.**
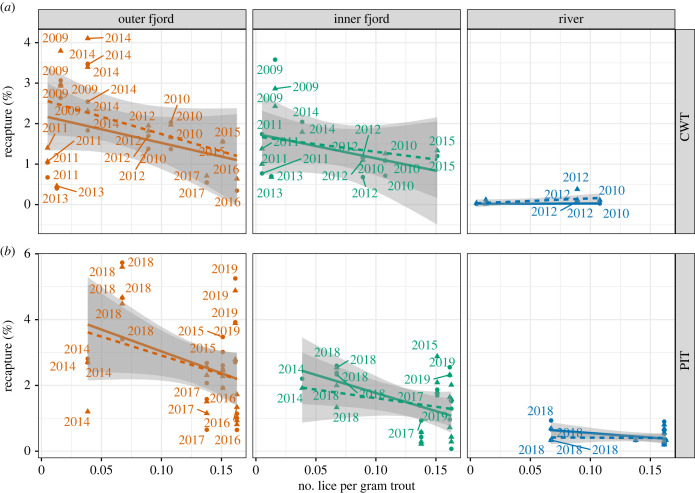
Relationship between recapture and number of lice per gram of trout in treated (solid line and circular dots) and control (untreated, dashed line and triangles) groups, divided into release sites. (*a*) Data from fish tagged with coded wired tags (CWTs); (*b*) data from fish tagged with passive integrated transponders (PITs). The lines and grey envelopes show linear fit to the data with 95% confidence interval.

**Figure 8.**
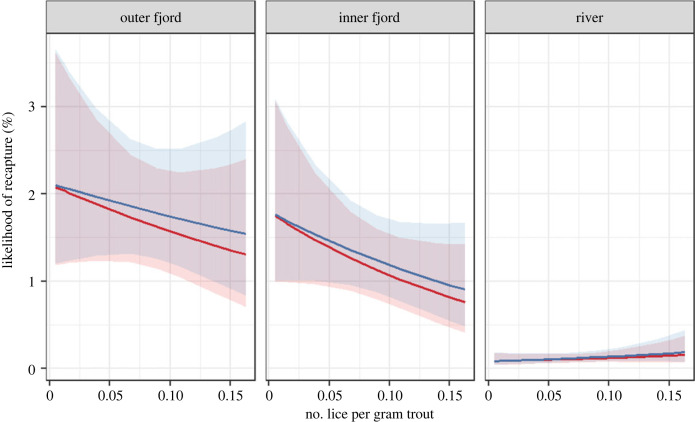
Modelled likelihood of recapture from lmer model where effect of release site and lice infestation (lice per gram trout) is visualized. Blue represents treated groups while red represents control (non-treated) groups. The envelopes indicate 95% confidence intervals from model.

The effect of lice levels on recapture of hatchery smolt followed a clear pattern, with a decreased recapture in years with high salmon lice observed on sea trout (table 2). These patterns are apparent in both the control and treatment groups for fish tagged with PITs and CWTs (table 2; figure 7). The full model of recapture rate included all parameters and explained approximately 24% of the variance in the data (table 2). The model suggested that recapture rate of adult salmon decreased with increasing lice levels among fish released at all locations, albeit with a different slope for the different locations (table 2; figure 8). Treated fish also had a decrease in recapture with increasing sea lice levels, but less so than control fish. Also notable, recapture in the inner fjord was lower than in the outer fjord (odds ratio = 0.68), and much lower in the river compared with the outer fjord (odds ratio = 0.07). Finally, recapture of PIT-tagged fish was higher than CWT-tagged fish (odds ratio = 1.38).

**Table 2.** Model coefficient of final model of recapture rate of adult salmon. lpg, number of lice per gram of trout.

predictor	recapture rate of adult salmon		
	odds ratio	CI	*p*
(intercept)	0.01	0.01–0.01	<0.001
lpg	0.74	0.53–1.05	0.092
treatment	1.46	1.37–1.56	<0.001
release location: inner fjord	0.11	0.08–0.15	<0.001
release location: outer fjord	1.11	1.05–1.17	<0.001
tag type: PIT	1.38	1.25–1.51	<0.001
lpg: inner fjord	1.14	1.07–1.21	<0.001
lpg: outer fjord	1.66	1.17–2.36	0.004
lpg: treatment	1.06	1.00–1.12	0.044
*random effects*			
*σ*^2^	3.29		
*τ*_00_ release_year	0.31		
*N*_release_year_	10		
no. observations	439 852		
marginal *R*^2^/conditional *R*^2^	0.242/0.242

### (e) Effect of infestation pressure on spawning count assessment

**Figure 9.**
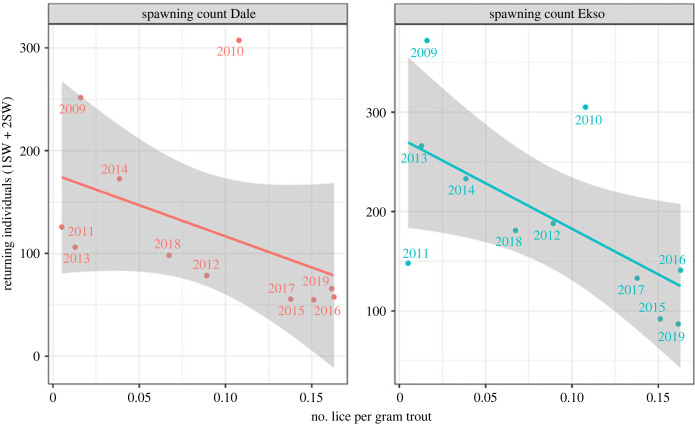
Correlation plot between number of returning individuals counted during spawning counts and number of lice per gram of fish weight on sea trout monitored in the out-migration route during the year of out-migration. The lines and grey envelopes show linear fit to the data and 95% confidence intervals.

There was a negative correlation between lice levels and spawning count assessments in the nearby rivers (figure 9), which was significant in Ekso, but not in Dale (Dale: *t* = −1.4725, d.f. = 9, *p*-value = 0.175, corr = −0.44; Ekso: *t* = −2.4266, d.f. = 9, *p*-value = 0.0382, corr = −0.63).

## 4. Discussion

RCTs aiming to estimate the impact of lice on wild salmon have apparently underestimated the effect of salmon lice on out-migrating salmon smolts, and suggest that the impact of salmon lice on wild salmon is larger than has previously been reported. This example of parasite spillback is one of the most studied host–parasite systems, and the results have direct management implications for anadromous salmonid populations. Our results exemplify the challenges of obtaining accurate assessments of parasite spillback effects, even for well-studied species such as Atlantic salmon with carefully curated experimental designs such as RCT.

There was a correlation between sea lice infestation pressure estimated from sea trout surveillance in the smolt migration route and the subsequent return rates of adult salmon, which we submit is a mechanistic correlation illustrating parasite spillback. This correlation suggests that from 2009 to 2018, years of low survival were driven by lice infestation pressure on out-migrating salmon smolts. However, the effect of treatment against salmon lice was only marginally modified by the lice levels ascertained from sentinel sea trout. Evidently, the anti-lice treatment only partially shielded the out-migrating salmon from sea lice during periods of high infestation pressure. This is in line with other recent studies suggesting that (i) salmon lice in western Norway are resistant to emamectin benzoate [31], (ii) the effect of treatment in RCTs is only significant and positive when infestation pressure is high [19], (iii) treatment may not be effective across all individuals in the group [20], and (iv) treatment may also incur negative effects on marine survival cancelling out the positive effect of the antiparasitic treatment on the return rate of salmon [21].

Vollset *et al.* [16,17] found a strong relationship between survival in the control group and the effect size of treatment. If treatment only incurs a benefit when impacts of sea lice are high, and potentially a negative effect when impacts of sea lice are low, one may also expect that the correlation between survival in the control group and the RR is driven by the effect of sea lice. This hypothesis suggests that survival of the control group should be correlated with the survival of infestation pressure from sea lice, and that RRs should be correlated with the same infestation pressure. Our results suggest exactly that; return rates were strongly correlated with how many salmon lice were observed on sea trout during the weeks after the release of salmon smolt.

A correlation between return rates of salmon and lice infestation pressure could be a result of a spurious correlation of interannual variation in the two variables. In fact, this uncertainty is what the RCT studies aim to mitigate by exploiting the statistical power of paired groups [32]. Interannual variation in survival can naturally vary extensively depending on marine conditions [33]. For example, studies have suggested that regime shifts in survival and growth have been driven by large-scale changes in oceanographic conditions, propagating bottom-up effects through food webs that salmon exploit at sea [34]. Our current knowledge about regime shifts and large-scale interannual variation in survival on the west coast of Norway is that there have been two major events that have substantially changed survival conditions, growth and age at return from sea. In 2005, a major drop in marine growth was observed in a large part of the North Atlantic basin [34], while in 2009 a parallel increase in survival was observed across a smaller region along the southwestern coast of Norway [6]. During the last 12 years, no major shifts in either growth or survival have been observed, although there has been clear interannual variation in survival during the most recent period. In our study, we show that the interannual variation in survival during this last period could in large part be explained by the levels of lice, as measured on sea trout in the migration route, during the out-migration of salmon smolt.

In addition to the main study river Vosso, the correlation in abundance of returning adult salmon with lice levels was also observed in the nearby rivers Dale and Ekso, where salmon smolts have to migrate to sea through the same fjord system. It is important to note that such correlations could be driven by factors other than a direct causal effect of salmon lice. For example, temperature or discharge conditions in the fjord system may have simultaneously impacted both smolt survival and salmon lice growth. Notwithstanding, the correlation does support the *a priori* hypothesis that salmon lice do impact the return rate of wild Atlantic salmon. For example Johnsen *et al*. [24] used a hydrodynamic model combined with an individual-based model to estimate that salmon lice emitted from fish farms in the region would incur mortality in the range of 10–50%, depending on the parameterization of the model and year.

The salmon lice on sea trout in this sampling has been shown to correlate strongly with the infestation pressure and biomass in surrounding fish farms [6]. The lice levels during the period 2009–2021 must be described as high, with the median number of lice varying from 2 to 78 lice per fish among years, and the median number of lice per gram varying from approximately 0.01 to 0.41 lice per gram of fish. These high infestation levels seen in most years are not normally observed in areas without fish farms [8]. The reduced return rates in salmon must be mainly attributed to the spillback effect of parasites originating on farmed fish. Although salmon lice were not counted directly on out-migrating salmon smolts, lice numbers on salmon smolts correlate with the number of salmon lice on sea trout [35], although lice numbers on salmon smolts are known to be lower than on trout. However, salmon smolts are also smaller than sea trout and will, therefore, tolerate lower numbers of salmon lice [5]. The exact number of lice that will lead to pathology and death in salmon is still highly uncertain [30], but a number of experimental studies have shown that salmon smolts start to physiologically respond to the parasite from very low levels [5], and that mortality increases with increasing levels of lice most likely from the first lice developing into a mobile and more virulent developmental stage. This effect is also likely to be context-dependent, where for example the virulence of lice is higher when temperature is higher [6,36]. Consequently, it seems evident that these high infestation levels that are observed in Nordhordland in western Norway are reducing the return rates of salmon.

Intensive farming on land and at sea has created massive animal host reserves for pathogenic species to spill back to wild populations. Direct evidence that diseases proliferating on farmed hosts actually affect wild conspecifics (or co-vulnerables, such as the case of bovine tuberculosis and badgers in the UK) is challenging to obtain. RCTs are considered state-of-the-art for ecological experimental designs aiming to understand the impact of domestic-origin pathogens such as salmon lice on wild fish, but we demonstrate the shortcomings of even these carefully designed approaches. Illuminating population-level effects of stressors such as pathogens really requires integrated time series such as those collected for Atlantic salmon smolt migrants and lice levels on sentinel sea trout presented here. The unique scale of these two datasets and the ability to combine them in one fjord system linked to a major river suffering from a population collapse [37] render this example a key point in the discussion about parasite-induced mortality and the impact of domesticated fish in open net-pens placed in the migratory route of imperilled wild species; such is the case for wild Atlantic salmon in Norway, which was recently added to the Red List of threatened species. Continuation of this time series will be important to informing ongoing management of the industry and the sensitive fjord environments that are used by the industry.

### (a) Management implications

To mitigate the environmental impacts while simultaneously allowing the salmonid fish farming industry to develop, a management system termed the 'traffic light system’ (TLS) has been implemented in Norway [6]. This system regulates the allowable biomass in 13 production zones in Norway based on whether environmental indicators signal a tolerable environmental impact or not. As of now, the effect of salmon lice on out-migrating wild salmon is the only environmental indicator that has been operationalized as part of the TLS, and thereby the only checkpoint for further growth of aquaculture in each production area is the effect of salmon lice on out-migrating wild salmon. The environmental indicator threshold has been defined as acceptable or unacceptable based on whether out-migrating salmon smolt survival is reduced by 10% (acceptable), 10–30% (intermediate) or more than 30% (unacceptable), based on 2 year cycles. When production areas achieve ‘acceptable’ status, 6% biomass increase is allowed, while a 6% reduction in biomass is required for areas deemed unacceptable. The share of the out-migrating salmon smolts that are likely to die owing to salmon lice is assessed based on surveys on salmon and trout (as in this study) and models that predict the number of lice on the salmon and how many are above a threshold level that is expected to lead to death in the wild [24,30,38]. The core assumption of the TLS is that the assessed reduction in survival during the smolt out-migration is linked to an eventual population-level effect on adults returning to the river to spawn—the key metric by which rivers are monitored in Norway. Therefore, the validity of the effect of sea lice as a credible environmental indicator must be assessed by linking return rates of adult salmon to sea lice infestation pressure on out-migrating salmon smolts. This was also the conclusion from an international evaluation committee that evaluated the TLS in 2021 [39]. This study is the first, to our knowledge, to link lice levels on sea trout and return rates of salmon, and simultaneously show that RCT studies do not provide unequivocal results that can be used to assess acceptable levels of salmon lice impacts on salmon populations.

## 5. Conclusion

Parasites are natural stressors in ecological systems. However, sea lice have reached epidemic proportions in Norwegian coastal waters. Lice have, therefore, become a stressor that affects population productivity. Despite this new designation, owing to the industrial production of fish being farmed in open sea cages, sea lice are not an isolated stressor. Atlantic salmon in Norway are facing urgent threats from multiple angles [9,21]. Multiple stressors in ecological systems are challenging to unravel because interactions can be synergistic, confounding management efforts. Because salmon spend the early part of their life in rivers, production can be limited by the habitat quantity and quality in the rivers. Indeed, Flávio *et al*. [40] suggested that a share of smolt mortality attributed to effects in the marine environment was actually occurring in the rivers during the out-migration. More research on these synergies between freshwater and marine stressors will be valuable. Parasite spillback is an emerging topic in conservation biology, and although spill-over effects (i.e. the introduction of new diseases from farms to wildlife or wildlife to humans; e.g. COVID-19) are often defined as more critical, spillback effects are most likely more widespread because of the nature of host–parasite dynamics. However, parasite spillback, and particularly intraspecific spillback, is generally more difficult to assess because it is often difficult to disentangle natural or climate-driven changes in parasite levels from that of presence of farmed animals. The example of wild salmon, salmon lice and farmed salmon exemplifies how difficult it can be to get accurate estimates even in well studied systems.

## Data Availability

All data are available from the Zenodo Digital Repository: https://doi.org/10.5281/zenodo.7081739 [41].
